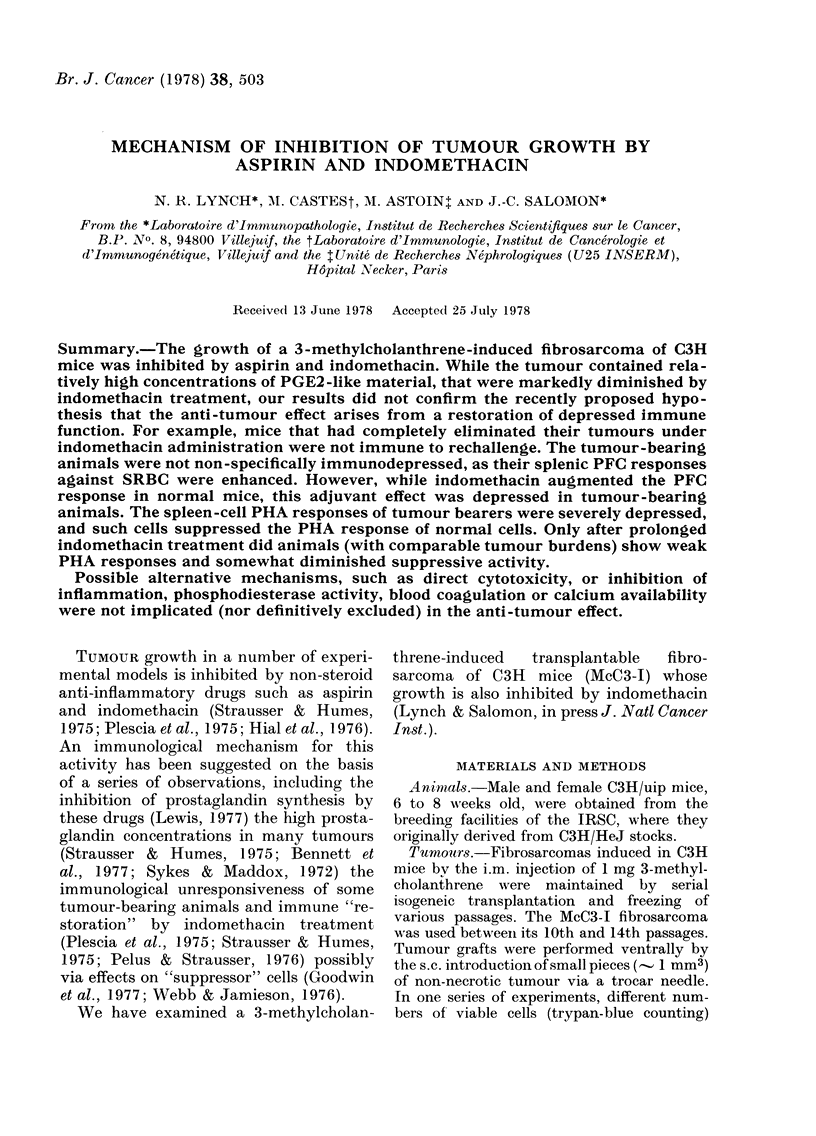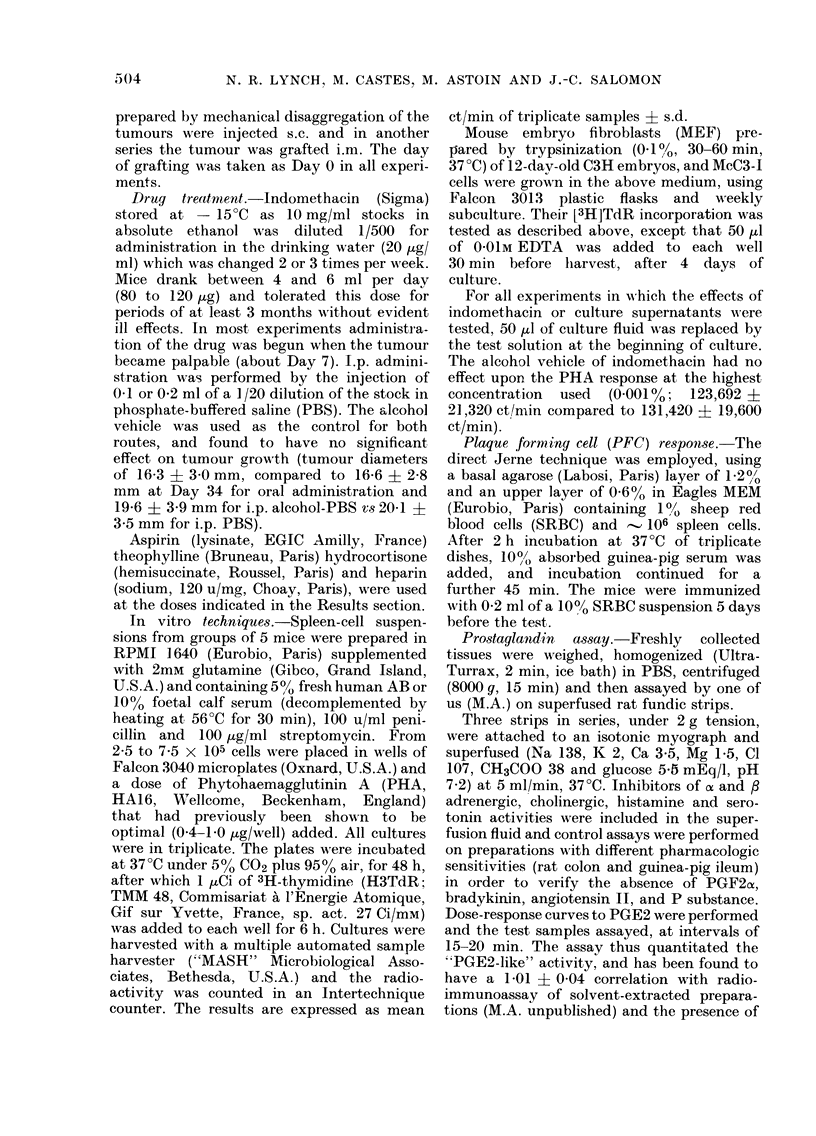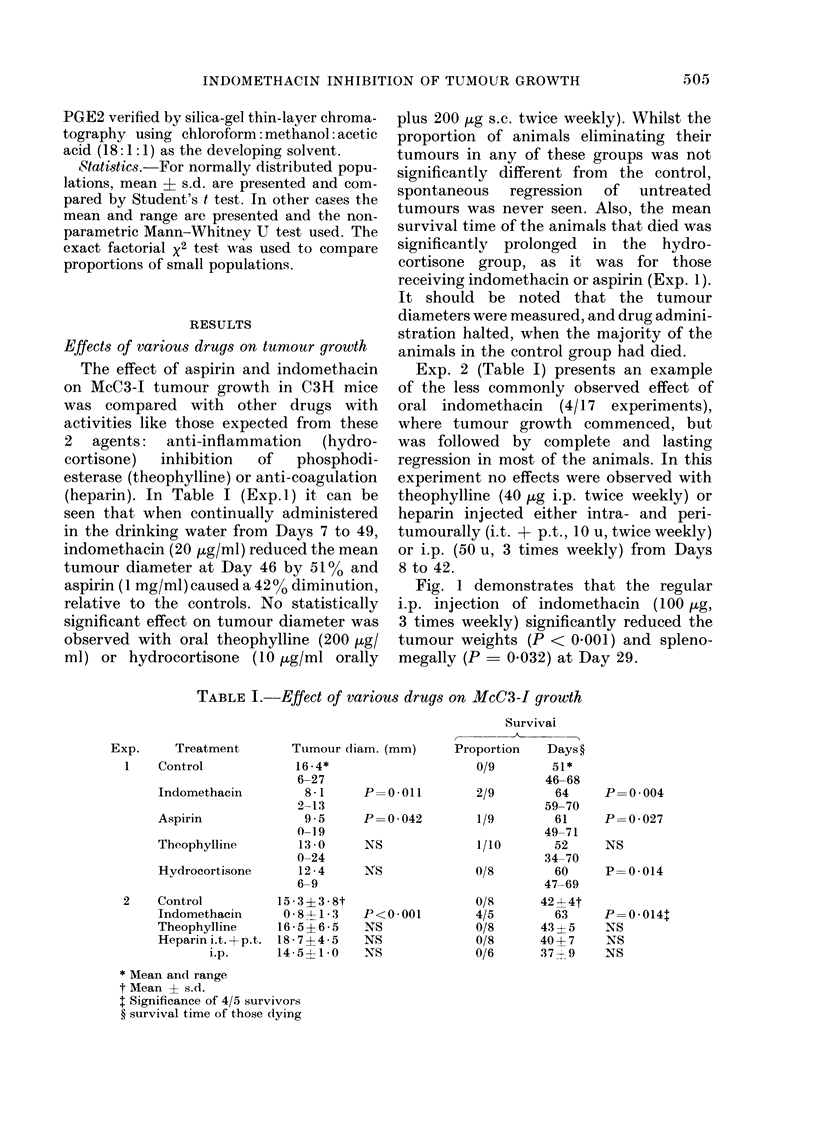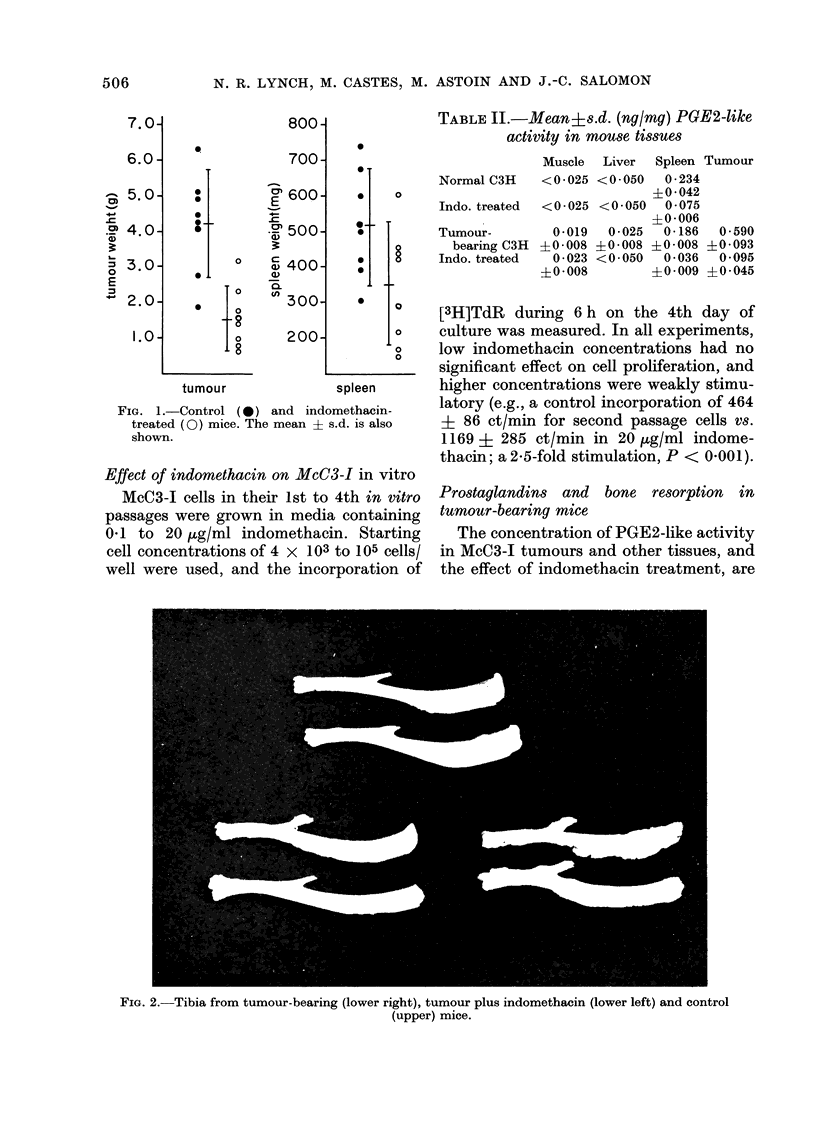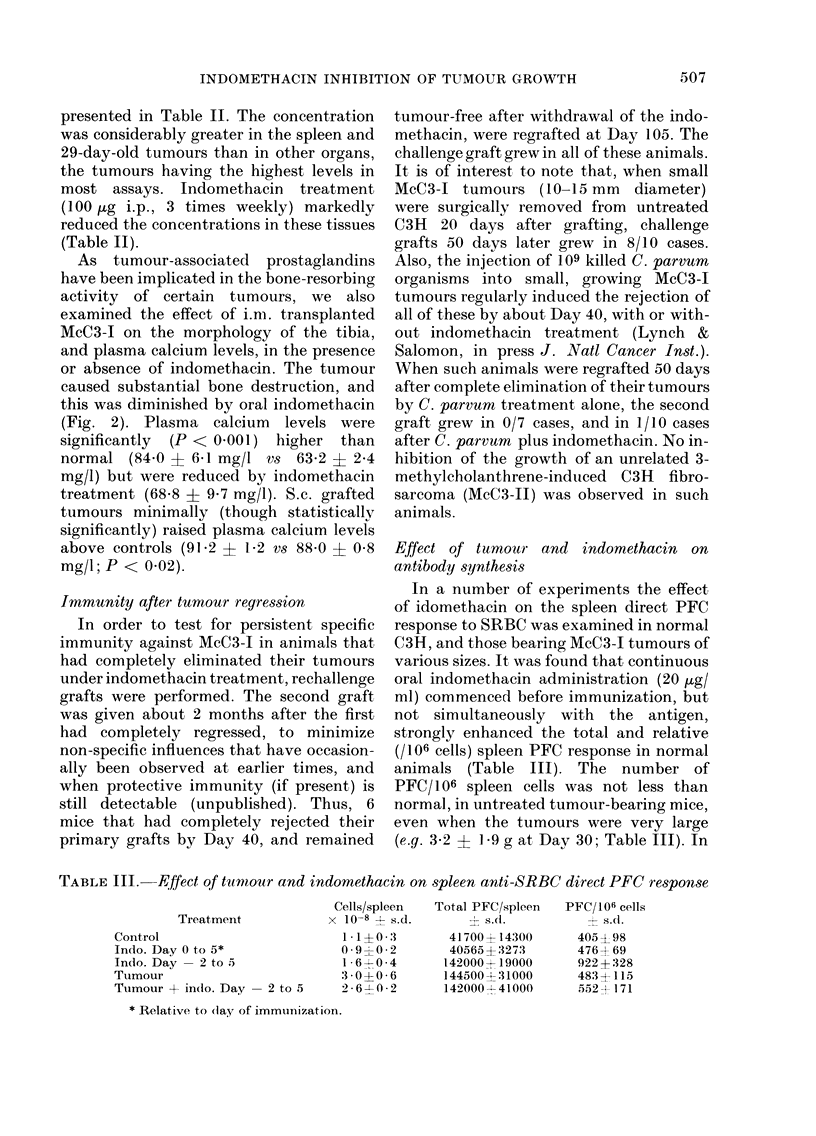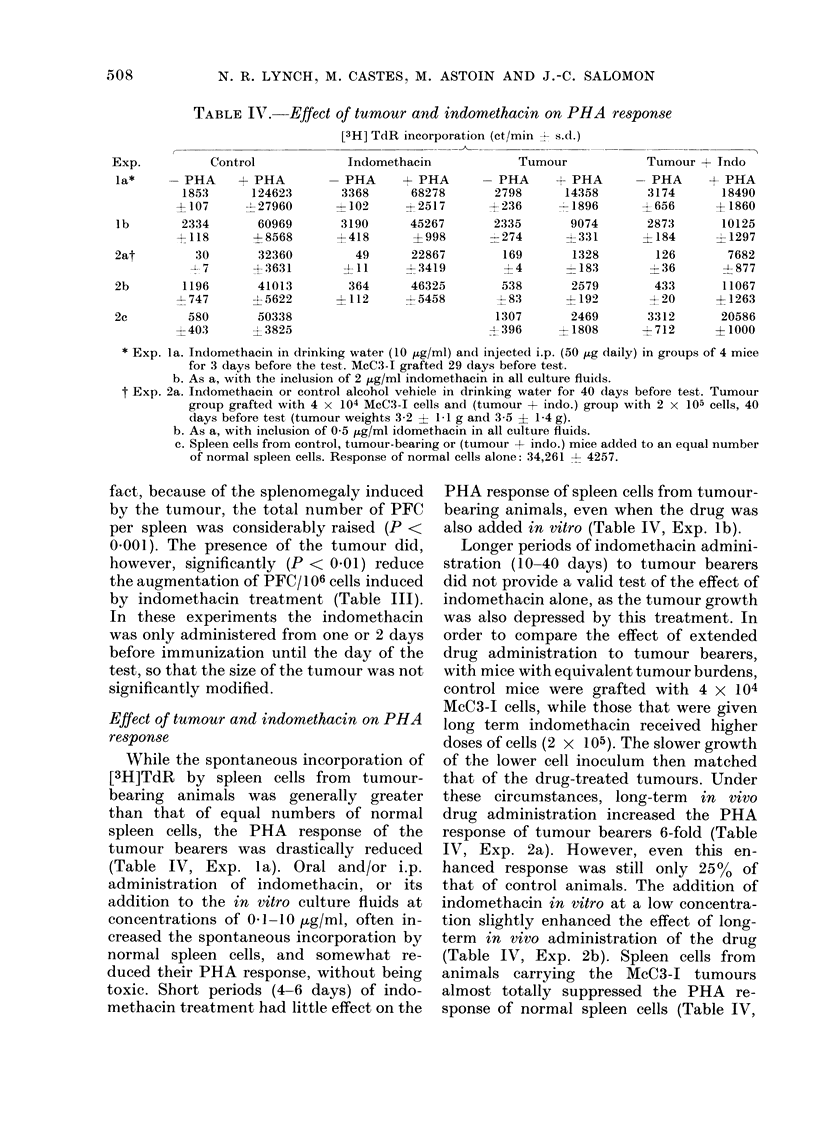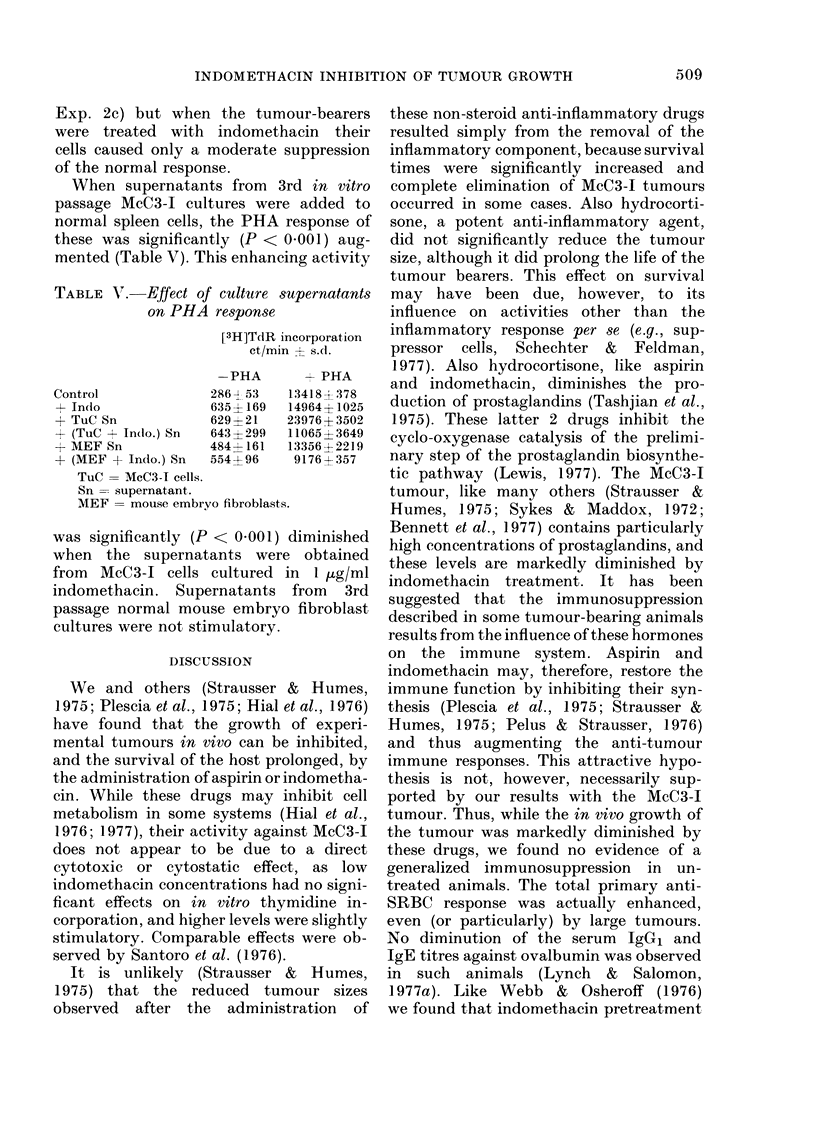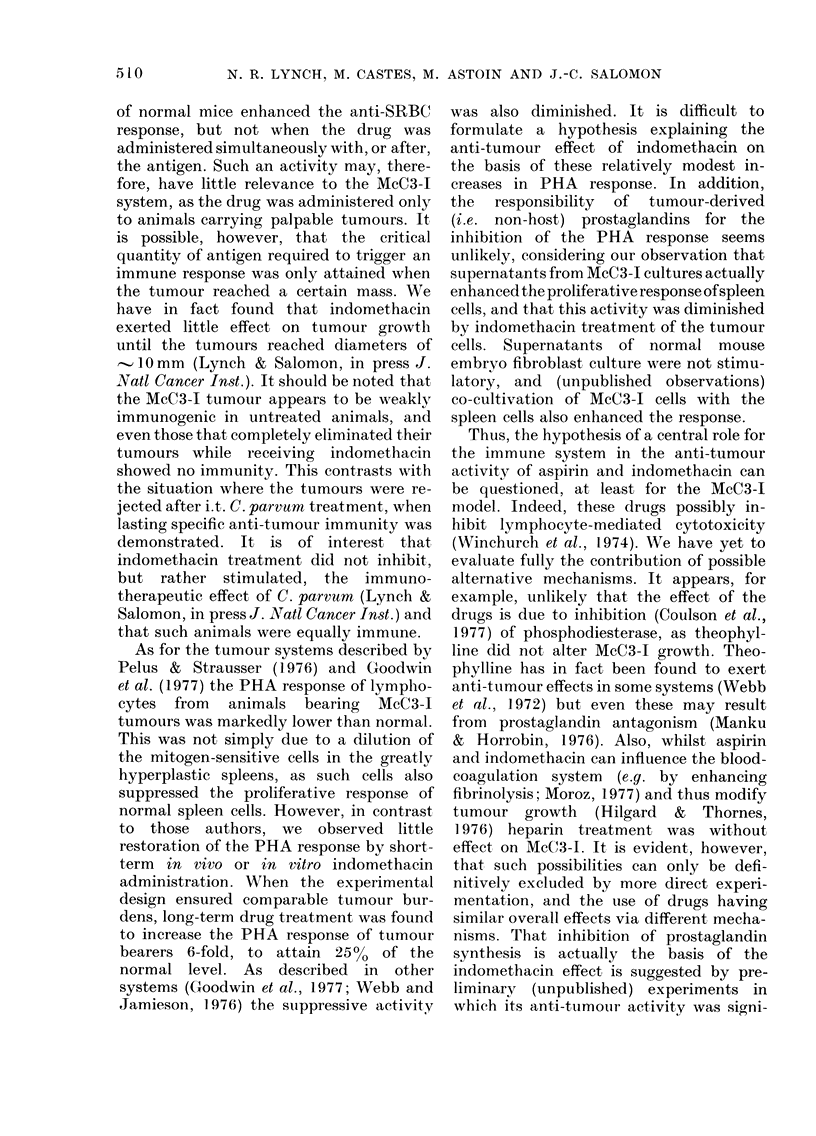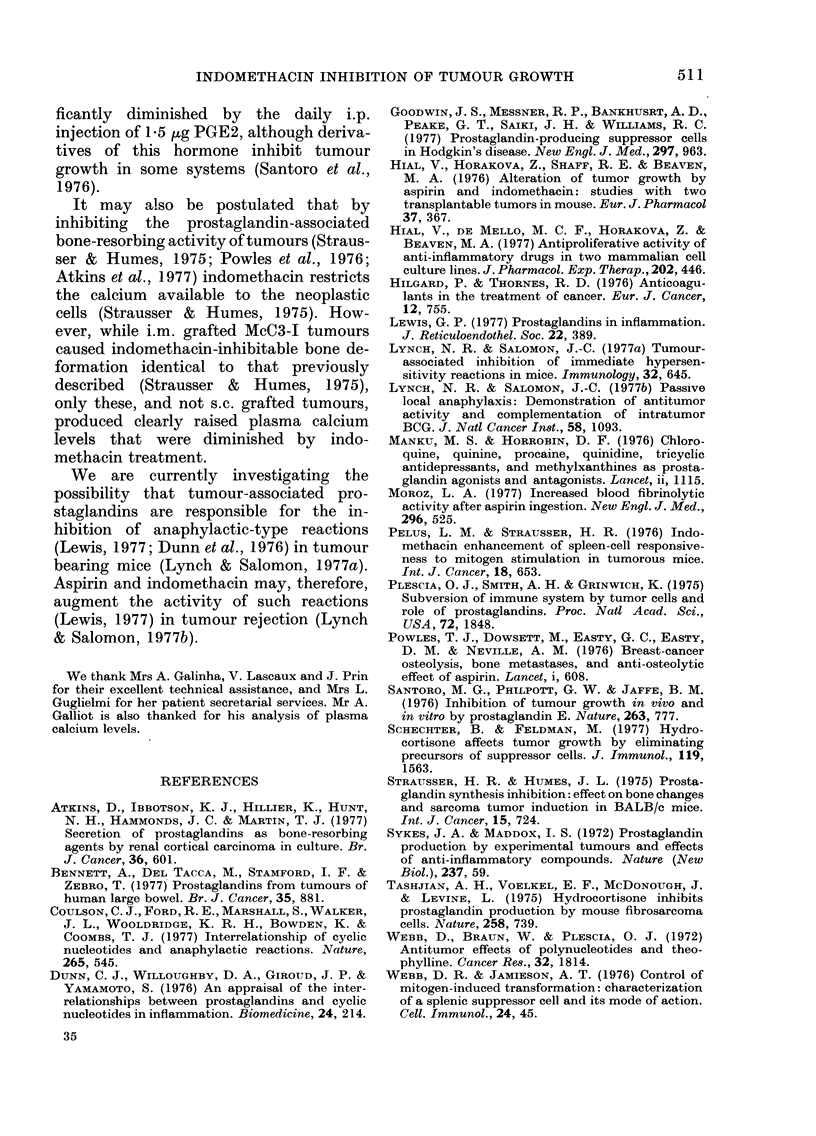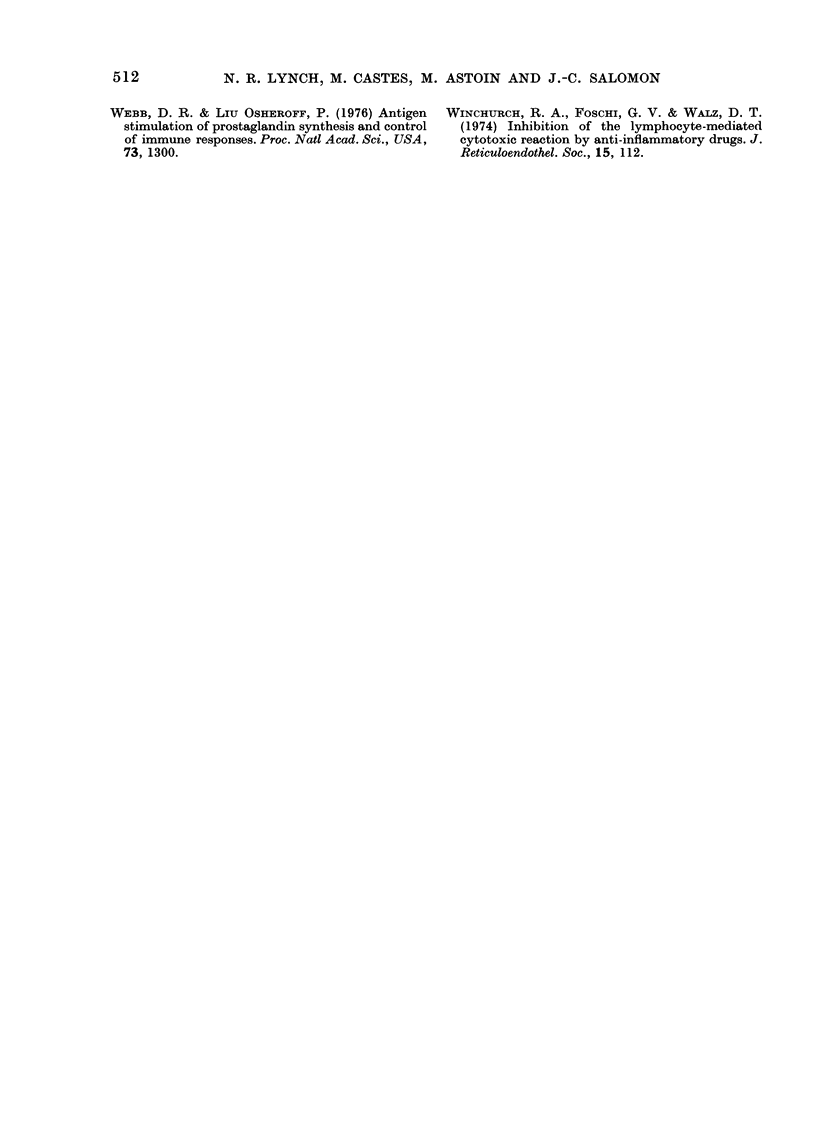# Mechanism of inhibition of tumour growth by aspirin and indomethacin.

**DOI:** 10.1038/bjc.1978.237

**Published:** 1978-10

**Authors:** N. R. Lynch, M. Castes, M. Astoin, J. C. Salomon

## Abstract

**Images:**


Br. J. Cancer (1978) 38, 503

### MECHANISM OF INHIBITION OF TUMOUR GROWTH BY ASPIRIN AND INDOMETHACIN

N. R. LYNCH*, M. CASTES†, M. ASTOIN‡ AND J.-C. SALOMON*

*From the *Laboratoire d'Immunopathologie, Institut de Recherches Scientifiques sur le Cancer, B.P. N°. 8, 94800 Villejuif, the †Laboratoire d'Immunologie, Institut de Cancérologie et d'Immunogénétique, Villejuif and the ‡Unité de Recherches Néphrologiques (U25 INSERM), Hôpital Necker, Paris*

Received 13 June 1978 Accepted 25 July 1978

**Summary.—The growth of a 3-methylcholanthrene-induced fibrosarcoma of C3H mice was inhibited by aspirin and indomethacin. While the tumour contained relatively high concentrations of PGE2-like material, that were markedly diminished by indomethacin treatment, our results did not confirm the recently proposed hypothesis that the anti-tumour effect arises from a restoration of depressed immune function. For example, mice that had completely eliminated their tumours under indomethacin administration were not immune to rechallenge. The tumour-bearing animals were not non-specifically immunodepressed, as their splenic PFC responses against SRBC were enhanced. However, while indomethacin augmented the PFC response in normal mice, this adjuvant effect was depressed in tumour-bearing animals. The spleen-cell PHA responses of tumour bearers were severely depressed, and such cells suppressed the PHA response of normal cells. Only after prolonged indomethacin treatment did animals (with comparable tumour burdens) show weak PHA responses and somewhat diminished suppressive activity.**

**Possible alternative mechanisms, such as direct cytotoxicity, or inhibition of inflammation, phosphodiesterase activity, blood coagulation or calcium availability were not implicated (nor definitively excluded) in the anti-tumour effect.**

Tumour growth in a number of experimental models is inhibited by non-steroid anti-inflammatory drugs such as aspirin and indomethacin (Strausser & Humes, 1975; Plescia *et al.*, 1975; Hial *et al.*, 1976). An immunological mechanism for this activity has been suggested on the basis of a series of observations, including the inhibition of prostaglandin synthesis by these drugs (Lewis, 1977) the high prostaglandin concentrations in many tumours (Strausser & Humes, 1975; Bennett *et al.*, 1977; Sykes & Maddox, 1972) the immunological unresponsiveness of some tumour-bearing animals and immune "restoration" by indomethacin treatment (Plescia *et al.*, 1975; Strausser & Humes, 1975; Pelus & Strausser, 1976) possibly via effects on "suppressor" cells (Goodwin *et al.*, 1977; Webb & Jamieson, 1976).

We have examined a 3-methylcholanthrene-induced transplantable fibrosarcoma of C3H mice (McC3-I) whose growth is also inhibited by indomethacin (Lynch & Salomon, in press *J. Natl Cancer Inst.*).

### MATERIALS AND METHODS

*Animals.*—Male and female C3H/uip mice, 6 to 8 weeks old, were obtained from the breeding facilities of the IRSC, where they originally derived from C3H/HeJ stocks.

*Tumours.*—Fibrosarcomas induced in C3H mice by the i.m. injection of 1 mg 3-methylcholanthrene were maintained by serial isogeneic transplantation and freezing of various passages. The McC3-I fibrosarcoma was used between its 10th and 14th passages. Tumour grafts were performed ventrally by the s.c. introduction of small pieces ($\sim$ 1 mm$^3$) of non-necrotic tumour via a trocar needle. In one series of experiments, different numbers of viable cells (trypan-blue counting)

prepared by mechanical disaggregation of the tumours were injected s.c. and in another series the tumour was grafted i.m. The day of grafting was taken as Day 0 in all experiments.

*Drug treatment.*—Indomethacin (Sigma) stored at $-15°C$ as 10 mg/ml stocks in absolute ethanol was diluted 1/500 for administration in the drinking water (20 $\mu$g/ml) which was changed 2 or 3 times per week. Mice drank between 4 and 6 ml per day (80 to 120 $\mu$g) and tolerated this dose for periods of at least 3 months without evident ill effects. In most experiments administration of the drug was begun when the tumour became palpable (about Day 7). I.p. administration was performed by the injection of 0·1 or 0·2 ml of a 1/20 dilution of the stock in phosphate-buffered saline (PBS). The alcohol vehicle was used as the control for both routes, and found to have no significant effect on tumour growth (tumour diameters of $16·3 \pm 3·0$ mm, compared to $16·6 \pm 2·8$ mm at Day 34 for oral administration and $19·6 \pm 3·9$ mm for i.p. alcohol-PBS *vs* $20·1 \pm 3·5$ mm for i.p. PBS).

Aspirin (lysinate, EGIC Amilly, France) theophylline (Bruneau, Paris) hydrocortisone (hemisuccinate, Roussel, Paris) and heparin (sodium, 120 u/mg, Choay, Paris), were used at the doses indicated in the Results section.

In vitro *techniques.*—Spleen-cell suspensions from groups of 5 mice were prepared in RPMI 1640 (Eurobio, Paris) supplemented with 2mM glutamine (Gibco, Grand Island, U.S.A.) and containing 5% fresh human AB or 10% foetal calf serum (decomplemented by heating at 56°C for 30 min), 100 u/ml penicillin and 100 $\mu$g/ml streptomycin. From $2·5$ to $7·5 \times 10^5$ cells were placed in wells of Falcon 3040 microplates (Oxnard, U.S.A.) and a dose of Phytohaemagglutinin A (PHA, HA16, Wellcome, Beckenham, England) that had previously been shown to be optimal (0·4–1·0 $\mu$g/well) added. All cultures were in triplicate. The plates were incubated at 37°C under 5% $CO_2$ plus 95% air, for 48 h, after which 1 $\mu$Ci of $^3$H-thymidine (H3TdR; TMM 48, Commisariat à l'Energie Atomique, Gif sur Yvette, France, sp. act. 27 Ci/mM) was added to each well for 6 h. Cultures were harvested with a multiple automated sample harvester ("MASH" Microbiological Associates, Bethesda, U.S.A.) and the radioactivity was counted in an Intertechnique counter. The results are expressed as mean ct/min of triplicate samples $\pm$ s.d.

Mouse embryo fibroblasts (MEF) prepared by trypsinization (0·1%, 30–60 min, 37°C) of 12-day-old C3H embryos, and McC3-I cells were grown in the above medium, using Falcon 3013 plastic flasks and weekly subculture. Their [$^3$H]TdR incorporation was tested as described above, except that 50 $\mu$l of 0·01M EDTA was added to each well 30 min before harvest, after 4 days of culture.

For all experiments in which the effects of indomethacin or culture supernatants were tested, 50 $\mu$l of culture fluid was replaced by the test solution at the beginning of culture. The alcohol vehicle of indomethacin had no effect upon the PHA response at the highest concentration used (0·001%; $123{,}692 \pm 21{,}320$ ct/min compared to $131{,}420 \pm 19{,}600$ ct/min).

*Plaque forming cell (PFC) response.*—The direct Jerne technique was employed, using a basal agarose (Labosi, Paris) layer of 1·2% and an upper layer of 0·6% in Eagles MEM (Eurobio, Paris) containing 1% sheep red blood cells (SRBC) and $\sim 10^6$ spleen cells. After 2 h incubation at 37°C of triplicate dishes, 10% absorbed guinea-pig serum was added, and incubation continued for a further 45 min. The mice were immunized with 0·2 ml of a 10% SRBC suspension 5 days before the test.

*Prostaglandin assay.*—Freshly collected tissues were weighed, homogenized (Ultra-Turrax, 2 min, ice bath) in PBS, centrifuged (8000 *g*, 15 min) and then assayed by one of us (M.A.) on superfused rat fundic strips.

Three strips in series, under 2 g tension, were attached to an isotonic myograph and superfused (Na 138, K 2, Ca 3·5, Mg 1·5, Cl 107, $CH_3COO$ 38 and glucose 5·5 mEq/l, pH 7·2) at 5 ml/min, 37°C. Inhibitors of $\alpha$ and $\beta$ adrenergic, cholinergic, histamine and serotonin activities were included in the superfusion fluid and control assays were performed on preparations with different pharmacologic sensitivities (rat colon and guinea-pig ileum) in order to verify the absence of PGF2$\alpha$, bradykinin, angiotensin II, and P substance. Dose-response curves to PGE2 were performed and the test samples assayed, at intervals of 15–20 min. The assay thus quantitated the "PGE2-like" activity, and has been found to have a $1·01 \pm 0·04$ correlation with radioimmunoassay of solvent-extracted preparations (M.A. unpublished) and the presence of

PGE2 verified by silica-gel thin-layer chromatography using chloroform:methanol:acetic acid (18:1:1) as the developing solvent.

*Statistics.*—For normally distributed populations, mean ± s.d. are presented and compared by Student's $t$ test. In other cases the mean and range are presented and the non-parametric Mann–Whitney U test used. The exact factorial $\chi^2$ test was used to compare proportions of small populations.

### RESULTS

### *Effects of various drugs on tumour growth*

The effect of aspirin and indomethacin on McC3-I tumour growth in C3H mice was compared with other drugs with activities like those expected from these 2 agents: anti-inflammation (hydrocortisone) inhibition of phosphodiesterase (theophylline) or anti-coagulation (heparin). In Table I (Exp.1) it can be seen that when continually administered in the drinking water from Days 7 to 49, indomethacin (20 μg/ml) reduced the mean tumour diameter at Day 46 by 51% and aspirin (1 mg/ml) caused a 42% diminution, relative to the controls. No statistically significant effect on tumour diameter was observed with oral theophylline (200 μg/ml) or hydrocortisone (10 μg/ml orally plus 200 μg s.c. twice weekly). Whilst the proportion of animals eliminating their tumours in any of these groups was not significantly different from the control, spontaneous regression of untreated tumours was never seen. Also, the mean survival time of the animals that died was significantly prolonged in the hydrocortisone group, as it was for those receiving indomethacin or aspirin (Exp. 1). It should be noted that the tumour diameters were measured, and drug administration halted, when the majority of the animals in the control group had died.

Exp. 2 (Table I) presents an example of the less commonly observed effect of oral indomethacin (4/17 experiments), where tumour growth commenced, but was followed by complete and lasting regression in most of the animals. In this experiment no effects were observed with theophylline (40 μg i.p. twice weekly) or heparin injected either intra- and peritumourally (i.t. + p.t., 10 u, twice weekly) or i.p. (50 u, 3 times weekly) from Days 8 to 42.

Fig. 1 demonstrates that the regular i.p. injection of indomethacin (100 μg, 3 times weekly) significantly reduced the tumour weights ($P < 0{\cdot}001$) and splenomegally ($P = 0{\cdot}032$) at Day 29.

TABLE I.—*Effect of various drugs on McC3-I growth*

| Exp. | Treatment | Tumour diam. (mm) | | Survival: Proportion | Survival: Days§ | |
|---|---|---|---|---|---|---|
| 1 | Control | 16·4* 6–27 | | 0/9 | 51* 46–68 | |
| | Indomethacin | 8·1 2–13 | P=0·011 | 2/9 | 64 59–70 | P=0·004 |
| | Aspirin | 9·5 0–19 | P=0·042 | 1/9 | 61 49–71 | P=0·027 |
| | Theophylline | 13·0 0–24 | NS | 1/10 | 52 34–70 | NS |
| | Hydrocortisone | 12·4 6–9 | NS | 0/8 | 60 47–69 | P=0·014 |
| 2 | Control | 15·3±3·8† | | 0/8 | 42±4† | |
| | Indomethacin | 0·8±1·3 | P<0·001 | 4/5 | 63 | P=0·014‡ |
| | Theophylline | 16·5±6·5 | NS | 0/8 | 43±5 | NS |
| | Heparin i.t.+p.t. | 18·7±4·5 | NS | 0/8 | 40±7 | NS |
| | i.p. | 14·5±1·0 | NS | 0/6 | 37±9 | NS |

* Mean and range

† Mean ± s.d.

‡ Significance of 4/5 survivors

§ survival time of those dying

FIG. 1.—Control (●) and indomethacin-treated (○) mice. The mean ± s.d. is also shown.

### *Effect of indomethacin on McC3-I* in vitro

McC3-I cells in their 1st to 4th *in vitro* passages were grown in media containing 0·1 to 20 μg/ml indomethacin. Starting cell concentrations of $4 \times 10^3$ to $10^5$ cells/well were used, and the incorporation of

TABLE II.—*Mean±s.d. (ng/mg) PGE2-like activity in mouse tissues*

| | Muscle | Liver | Spleen | Tumour |
|---|---|---|---|---|
| Normal C3H | <0·025 | <0·050 | 0·234 ±0·042 | |
| Indo. treated | <0·025 | <0·050 | 0·075 ±0·006 | |
| Tumour-bearing C3H | 0·019 ±0·008 | 0·025 ±0·008 | 0·186 ±0·008 | 0·590 ±0·093 |
| Indo. treated | 0·023 ±0·008 | <0·050 | 0·036 ±0·009 | 0·095 ±0·045 |

[$^3$H]TdR during 6 h on the 4th day of culture was measured. In all experiments, low indomethacin concentrations had no significant effect on cell proliferation, and higher concentrations were weakly stimulatory (e.g., a control incorporation of 464 ± 86 ct/min for second passage cells *vs.* 1169 ± 285 ct/min in 20 μg/ml indomethacin; a 2·5-fold stimulation, $P < 0{\cdot}001$).

### *Prostaglandins and bone resorption in tumour-bearing mice*

The concentration of PGE2-like activity in McC3-I tumours and other tissues, and the effect of indomethacin treatment, are

FIG. 2.—Tibia from tumour-bearing (lower right), tumour plus indomethacin (lower left) and control (upper) mice.

presented in Table II. The concentration was considerably greater in the spleen and 29-day-old tumours than in other organs, the tumours having the highest levels in most assays. Indomethacin treatment (100 $\mu$g i.p., 3 times weekly) markedly reduced the concentrations in these tissues (Table II).

As tumour-associated prostaglandins have been implicated in the bone-resorbing activity of certain tumours, we also examined the effect of i.m. transplanted McC3-I on the morphology of the tibia, and plasma calcium levels, in the presence or absence of indomethacin. The tumour caused substantial bone destruction, and this was diminished by oral indomethacin (Fig. 2). Plasma calcium levels were significantly ($P < 0{\cdot}001$) higher than normal ($84{\cdot}0 \pm 6{\cdot}1$ mg/l *vs* $63{\cdot}2 \pm 2{\cdot}4$ mg/l) but were reduced by indomethacin treatment ($68{\cdot}8 \pm 9{\cdot}7$ mg/l). S.c. grafted tumours minimally (though statistically significantly) raised plasma calcium levels above controls ($91{\cdot}2 \pm 1{\cdot}2$ *vs* $88{\cdot}0 \pm 0{\cdot}8$ mg/l; $P < 0{\cdot}02$).

*Immunity after tumour regression*

In order to test for persistent specific immunity against McC3-I in animals that had completely eliminated their tumours under indomethacin treatment, rechallenge grafts were performed. The second graft was given about 2 months after the first had completely regressed, to minimize non-specific influences that have occasionally been observed at earlier times, and when protective immunity (if present) is still detectable (unpublished). Thus, 6 mice that had completely rejected their primary grafts by Day 40, and remained tumour-free after withdrawal of the indomethacin, were regrafted at Day 105. The challenge graft grew in all of these animals. It is of interest to note that, when small McC3-I tumours (10–15 mm diameter) were surgically removed from untreated C3H 20 days after grafting, challenge grafts 50 days later grew in 8/10 cases. Also, the injection of $10^9$ killed *C. parvum* organisms into small, growing McC3-I tumours regularly induced the rejection of all of these by about Day 40, with or without indomethacin treatment (Lynch & Salomon, in press *J. Natl Cancer Inst.*). When such animals were regrafted 50 days after complete elimination of their tumours by *C. parvum* treatment alone, the second graft grew in 0/7 cases, and in 1/10 cases after *C. parvum* plus indomethacin. No inhibition of the growth of an unrelated 3-methylcholanthrene-induced C3H fibrosarcoma (McC3-II) was observed in such animals.

*Effect of tumour and indomethacin on antibody synthesis*

In a number of experiments the effect of idomethacin on the spleen direct PFC response to SRBC was examined in normal C3H, and those bearing McC3-I tumours of various sizes. It was found that continuous oral indomethacin administration (20 $\mu$g/ml) commenced before immunization, but not simultaneously with the antigen, strongly enhanced the total and relative (/$10^6$ cells) spleen PFC response in normal animals (Table III). The number of PFC/$10^6$ spleen cells was not less than normal, in untreated tumour-bearing mice, even when the tumours were very large (*e.g.* $3{\cdot}2 \pm 1{\cdot}9$ g at Day 30; Table III). In

TABLE III.—*Effect of tumour and indomethacin on spleen anti-SRBC direct PFC response*

| Treatment | Cells/spleen $\times 10^{-8} \pm$ s.d. | Total PFC/spleen $\pm$ s.d. | PFC/$10^6$ cells $\pm$ s.d. |
|---|---|---|---|
| Control | $1{\cdot}1 \pm 0{\cdot}3$ | $41700 \pm 14300$ | $405 \pm 98$ |
| Indo. Day 0 to 5* | $0{\cdot}9 \pm 0{\cdot}2$ | $40565 \pm 3273$ | $476 \pm 69$ |
| Indo. Day − 2 to 5 | $1{\cdot}6 \pm 0{\cdot}4$ | $142000 \pm 19000$ | $922 \pm 328$ |
| Tumour | $3{\cdot}0 \pm 0{\cdot}6$ | $144500 \pm 31000$ | $483 \pm 115$ |
| Tumour + indo. Day − 2 to 5 | $2{\cdot}6 \pm 0{\cdot}2$ | $142000 \pm 41000$ | $552 \pm 171$ |

* Relative to day of immunization.

TABLE IV.—*Effect of tumour and indomethacin on PHA response*

| Exp. | Control | | Indomethacin | | Tumour | | Tumour + Indo | |
|---|---|---|---|---|---|---|---|---|
| | [³H] TdR incorporation (ct/min ± s.d.) | | | | | | | |
| | − PHA | + PHA | − PHA | + PHA | − PHA | + PHA | − PHA | + PHA |
| 1a* | 1853 ±107 | 124623 ±27960 | 3368 ±102 | 68278 ±2517 | 2798 ±236 | 14358 ±1896 | 3174 ±656 | 18490 ±1860 |
| 1b | 2334 ±118 | 60969 ±8568 | 3190 ±418 | 45267 ±998 | 2335 ±274 | 9074 ±331 | 2873 ±184 | 10125 ±1297 |
| 2a† | 30 ±7 | 32360 ±3631 | 49 ±11 | 22867 ±3419 | 169 ±4 | 1328 ±183 | 126 ±36 | 7682 ±877 |
| 2b | 1196 ±747 | 41013 ±5622 | 364 ±112 | 46325 ±5458 | 538 ±83 | 2579 ±192 | 433 ±20 | 11067 ±1263 |
| 2c | 580 ±403 | 50338 ±3825 | | | 1307 ±396 | 2469 ±1808 | 3312 ±712 | 20586 ±1000 |

* Exp. 1a. Indomethacin in drinking water (10 μg/ml) and injected i.p. (50 μg daily) in groups of 4 mice for 3 days before the test. McC3-I grafted 29 days before test.
b. As a, with the inclusion of 2 μg/ml indomethacin in all culture fluids.

† Exp. 2a. Indomethacin or control alcohol vehicle in drinking water for 40 days before test. Tumour group grafted with $4 \times 10^4$ McC3-I cells and (tumour + indo.) group with $2 \times 10^5$ cells, 40 days before test (tumour weights 3·2 ± 1·1 g and 3·5 ± 1·4 g).
b. As a, with inclusion of 0·5 μg/ml idomethacin in all culture fluids.
c. Spleen cells from control, tumour-bearing or (tumour + indo.) mice added to an equal number of normal spleen cells. Response of normal cells alone: 34,261 ± 4257.

fact, because of the splenomegaly induced by the tumour, the total number of PFC per spleen was considerably raised ($P <$ 0·001). The presence of the tumour did, however, significantly ($P <$ 0·01) reduce the augmentation of PFC/$10^6$ cells induced by indomethacin treatment (Table III). In these experiments the indomethacin was only administered from one or 2 days before immunization until the day of the test, so that the size of the tumour was not significantly modified.

*Effect of tumour and indomethacin on PHA response*

While the spontaneous incorporation of [³H]TdR by spleen cells from tumour-bearing animals was generally greater than that of equal numbers of normal spleen cells, the PHA response of the tumour bearers was drastically reduced (Table IV, Exp. 1a). Oral and/or i.p. administration of indomethacin, or its addition to the *in vitro* culture fluids at concentrations of 0·1–10 μg/ml, often increased the spontaneous incorporation by normal spleen cells, and somewhat reduced their PHA response, without being toxic. Short periods (4–6 days) of indomethacin treatment had little effect on the PHA response of spleen cells from tumour-bearing animals, even when the drug was also added *in vitro* (Table IV, Exp. 1b).

Longer periods of indomethacin administration (10–40 days) to tumour bearers did not provide a valid test of the effect of indomethacin alone, as the tumour growth was also depressed by this treatment. In order to compare the effect of extended drug administration to tumour bearers, with mice with equivalent tumour burdens, control mice were grafted with $4 \times 10^4$ McC3-I cells, while those that were given long term indomethacin received higher doses of cells ($2 \times 10^5$). The slower growth of the lower cell inoculum then matched that of the drug-treated tumours. Under these circumstances, long-term *in vivo* drug administration increased the PHA response of tumour bearers 6-fold (Table IV, Exp. 2a). However, even this enhanced response was still only 25% of that of control animals. The addition of indomethacin *in vitro* at a low concentration slightly enhanced the effect of long-term *in vivo* administration of the drug (Table IV, Exp. 2b). Spleen cells from animals carrying the McC3-I tumours almost totally suppressed the PHA response of normal spleen cells (Table IV,

Exp. 2c) but when the tumour-bearers were treated with indomethacin their cells caused only a moderate suppression of the normal response.

When supernatants from 3rd *in vitro* passage McC3-I cultures were added to normal spleen cells, the PHA response of these was significantly ($P < 0{\cdot}001$) augmented (Table V). This enhancing activity

TABLE V.—*Effect of culture supernatants on PHA response*

| | [$^3$H]TdR incorporation ct/min ± s.d. | |
|---|---|---|
| | −PHA | + PHA |
| Control | 286±53 | 13418±378 |
| + Indo | 635±169 | 14964±1025 |
| + TuC Sn | 629±21 | 23976±3502 |
| + (TuC + Indo.) Sn | 643±299 | 11065±3649 |
| + MEF Sn | 484±161 | 13356±2219 |
| + (MEF + Indo.) Sn | 554±96 | 9176±357 |

TuC = McC3-I cells.
Sn = supernatant.
MEF = mouse embryo fibroblasts.

was significantly ($P < 0{\cdot}001$) diminished when the supernatants were obtained from McC3-I cells cultured in 1 μg/ml indomethacin. Supernatants from 3rd passage normal mouse embryo fibroblast cultures were not stimulatory.

### DISCUSSION

We and others (Strausser & Humes, 1975; Plescia *et al.*, 1975; Hial *et al.*, 1976) have found that the growth of experimental tumours *in vivo* can be inhibited, and the survival of the host prolonged, by the administration of aspirin or indomethacin. While these drugs may inhibit cell metabolism in some systems (Hial *et al.*, 1976; 1977), their activity against McC3-I does not appear to be due to a direct cytotoxic or cytostatic effect, as low indomethacin concentrations had no significant effects on *in vitro* thymidine incorporation, and higher levels were slightly stimulatory. Comparable effects were observed by Santoro *et al.* (1976).

It is unlikely (Strausser & Humes, 1975) that the reduced tumour sizes observed after the administration of these non-steroid anti-inflammatory drugs resulted simply from the removal of the inflammatory component, because survival times were significantly increased and complete elimination of McC3-I tumours occurred in some cases. Also hydrocortisone, a potent anti-inflammatory agent, did not significantly reduce the tumour size, although it did prolong the life of the tumour bearers. This effect on survival may have been due, however, to its influence on activities other than the inflammatory response *per se* (*e.g.*, suppressor cells, Schechter & Feldman, 1977). Also hydrocortisone, like aspirin and indomethacin, diminishes the production of prostaglandins (Tashjian *et al.*, 1975). These latter 2 drugs inhibit the cyclo-oxygenase catalysis of the preliminary step of the prostaglandin biosynthetic pathway (Lewis, 1977). The McC3-I tumour, like many others (Strausser & Humes, 1975; Sykes & Maddox, 1972; Bennett *et al.*, 1977) contains particularly high concentrations of prostaglandins, and these levels are markedly diminished by indomethacin treatment. It has been suggested that the immunosuppression described in some tumour-bearing animals results from the influence of these hormones on the immune system. Aspirin and indomethacin may, therefore, restore the immune function by inhibiting their synthesis (Plescia *et al.*, 1975; Strausser & Humes, 1975; Pelus & Strausser, 1976) and thus augmenting the anti-tumour immune responses. This attractive hypothesis is not, however, necessarily supported by our results with the McC3-I tumour. Thus, while the *in vivo* growth of the tumour was markedly diminished by these drugs, we found no evidence of a generalized immunosuppression in untreated animals. The total primary anti-SRBC response was actually enhanced, even (or particularly) by large tumours. No diminution of the serum $IgG_1$ and IgE titres against ovalbumin was observed in such animals (Lynch & Salomon, 1977*a*). Like Webb & Osheroff (1976) we found that indomethacin pretreatment of normal mice enhanced the anti-SRBC response, but not when the drug was administered simultaneously with, or after, the antigen. Such an activity may, therefore, have little relevance to the McC3-I system, as the drug was administered only to animals carrying palpable tumours. It is possible, however, that the critical quantity of antigen required to trigger an immune response was only attained when the tumour reached a certain mass. We have in fact found that indomethacin exerted little effect on tumour growth until the tumours reached diameters of $\sim$10 mm (Lynch & Salomon, in press *J. Natl Cancer Inst.*). It should be noted that the McC3-I tumour appears to be weakly immunogenic in untreated animals, and even those that completely eliminated their tumours while receiving indomethacin showed no immunity. This contrasts with the situation where the tumours were rejected after i.t. *C. parvum* treatment, when lasting specific anti-tumour immunity was demonstrated. It is of interest that indomethacin treatment did not inhibit, but rather stimulated, the immunotherapeutic effect of *C. parvum* (Lynch & Salomon, in press *J. Natl Cancer Inst.*) and that such animals were equally immune.

As for the tumour systems described by Pelus & Strausser (1976) and Goodwin *et al.* (1977) the PHA response of lymphocytes from animals bearing McC3-I tumours was markedly lower than normal. This was not simply due to a dilution of the mitogen-sensitive cells in the greatly hyperplastic spleens, as such cells also suppressed the proliferative response of normal spleen cells. However, in contrast to those authors, we observed little restoration of the PHA response by short-term *in vivo* or *in vitro* indomethacin administration. When the experimental design ensured comparable tumour burdens, long-term drug treatment was found to increase the PHA response of tumour bearers 6-fold, to attain 25% of the normal level. As described in other systems (Goodwin *et al.*, 1977; Webb and Jamieson, 1976) the suppressive activity was also diminished. It is difficult to formulate a hypothesis explaining the anti-tumour effect of indomethacin on the basis of these relatively modest increases in PHA response. In addition, the responsibility of tumour-derived (*i.e.* non-host) prostaglandins for the inhibition of the PHA response seems unlikely, considering our observation that supernatants from McC3-I cultures actually enhanced the proliferative response of spleen cells, and that this activity was diminished by indomethacin treatment of the tumour cells. Supernatants of normal mouse embryo fibroblast culture were not stimulatory, and (unpublished observations) co-cultivation of McC3-I cells with the spleen cells also enhanced the response.

Thus, the hypothesis of a central role for the immune system in the anti-tumour activity of aspirin and indomethacin can be questioned, at least for the McC3-I model. Indeed, these drugs possibly inhibit lymphocyte-mediated cytotoxicity (Winchurch *et al.*, 1974). We have yet to evaluate fully the contribution of possible alternative mechanisms. It appears, for example, unlikely that the effect of the drugs is due to inhibition (Coulson *et al.*, 1977) of phosphodiesterase, as theophylline did not alter McC3-I growth. Theophylline has in fact been found to exert anti-tumour effects in some systems (Webb *et al.*, 1972) but even these may result from prostaglandin antagonism (Manku & Horrobin, 1976). Also, whilst aspirin and indomethacin can influence the blood-coagulation system (*e.g.* by enhancing fibrinolysis; Moroz, 1977) and thus modify tumour growth (Hilgard & Thornes, 1976) heparin treatment was without effect on McC3-I. It is evident, however, that such possibilities can only be definitively excluded by more direct experimentation, and the use of drugs having similar overall effects via different mechanisms. That inhibition of prostaglandin synthesis is actually the basis of the indomethacin effect is suggested by preliminary (unpublished) experiments in which its anti-tumour activity was significantly diminished by the daily i.p. injection of 1·5 μg PGE2, although derivatives of this hormone inhibit tumour growth in some systems (Santoro *et al.*, 1976).

It may also be postulated that by inhibiting the prostaglandin-associated bone-resorbing activity of tumours (Strausser & Humes, 1975; Powles *et al.*, 1976; Atkins *et al.*, 1977) indomethacin restricts the calcium available to the neoplastic cells (Strausser & Humes, 1975). However, while i.m. grafted McC3-I tumours caused indomethacin-inhibitable bone deformation identical to that previously described (Strausser & Humes, 1975), only these, and not s.c. grafted tumours, produced clearly raised plasma calcium levels that were diminished by indomethacin treatment.

We are currently investigating the possibility that tumour-associated prostaglandins are responsible for the inhibition of anaphylactic-type reactions (Lewis, 1977; Dunn *et al.*, 1976) in tumour bearing mice (Lynch & Salomon, 1977*a*). Aspirin and indomethacin may, therefore, augment the activity of such reactions (Lewis, 1977) in tumour rejection (Lynch & Salomon, 1977*b*).

We thank Mrs A. Galinha, V. Lascaux and J. Prin for their excellent technical assistance, and Mrs L. Guglielmi for her patient secretarial services. Mr A. Galliot is also thanked for his analysis of plasma calcium levels.

### REFERENCES

ATKINS, D., IBBOTSON, K. J., HILLIER, K., HUNT, N. H., HAMMONDS, J. C. & MARTIN, T. J. (1977) Secretion of prostaglandins as bone-resorbing agents by renal cortical carcinoma in culture. *Br. J. Cancer*, **36**, 601.

BENNETT, A., DEL TACCA, M., STAMFORD, I. F. & ZEBRO, T. (1977) Prostaglandins from tumours of human large bowel. *Br. J. Cancer*, **35**, 881.

COULSON, C. J., FORD, R. E., MARSHALL, S., WALKER, J. L., WOOLDRIDGE, K. R. H., BOWDEN, K. & COOMBS, T. J. (1977) Interrelationship of cyclic nucleotides and anaphylactic reactions. *Nature*, **265**, 545.

DUNN, C. J., WILLOUGHBY, D. A., GIROUD, J. P. & YAMAMOTO, S. (1976) An appraisal of the interrelationships between prostaglandins and cyclic nucleotides in inflammation. *Biomedicine*, **24**, 214.

GOODWIN, J. S., MESSNER, R. P., BANKHUSRT, A. D., PEAKE, G. T., SAIKI, J. H. & WILLIAMS, R. C. (1977) Prostaglandin-producing suppressor cells in Hodgkin's disease. *New Engl. J. Med.*, **297**, 963.

HIAL, V., HORAKOVA, Z., SHAFF, R. E. & BEAVEN, M. A. (1976) Alteration of tumor growth by aspirin and indomethacin: studies with two transplantable tumors in mouse. *Eur. J. Pharmacol* **37**, 367.

HIAL, V., DE MELLO, M. C. F., HORAKOVA, Z. & BEAVEN, M. A. (1977) Antiproliferative activity of anti-inflammatory drugs in two mammalian cell culture lines. *J. Pharmacol. Exp. Therap.*, **202**, 446.

HILGARD, P. & THORNES, R. D. (1976) Anticoagulants in the treatment of cancer. *Eur. J. Cancer*, **12**, 755.

LEWIS, G. P. (1977) Prostaglandins in inflammation. *J. Reticuloendothel. Soc.* **22**, 389.

LYNCH, N. R. & SALOMON, J.-C. (1977*a*) Tumour-associated inhibition of immediate hypersensitivity reactions in mice. *Immunology*, **32**, 645.

LYNCH, N. R. & SALOMON, J.-C. (1977*b*) Passive local anaphylaxis: Demonstration of antitumor activity and complementation of intratumor BCG. *J. Natl Cancer Inst.*, **58**, 1093.

MANKU, M. S. & HORROBIN, D. F. (1976) Chloroquine, quinine, procaine, quinidine, tricyclic antidepressants, and methylxanthines as prostaglandin agonists and antagonists. *Lancet*, ii, 1115.

MOROZ, L. A. (1977) Increased blood fibrinolytic activity after aspirin ingestion. *New Engl. J. Med.*, **296**, 525.

PELUS, L. M. & STRAUSSER, H. R. (1976) Indomethacin enhancement of spleen-cell responsiveness to mitogen stimulation in tumorous mice. *Int. J. Cancer*, **18**, 653.

PLESCIA, O. J., SMITH, A. H. & GRINWICH, K. (1975) Subversion of immune system by tumor cells and role of prostaglandins. *Proc. Natl Acad. Sci., USA*, **72**, 1848.

POWLES, T. J., DOWSETT, M., EASTY, G. C., EASTY, D. M. & NEVILLE, A. M. (1976) Breast-cancer osteolysis, bone metastases, and anti-osteolytic effect of aspirin. *Lancet*, i, 608.

SANTORO, M. G., PHILPOTT, G. W. & JAFFE, B. M. (1976) Inhibition of tumour growth *in vivo* and *in vitro* by prostaglandin E. *Nature*, **263**, 777.

SCHECHTER, B. & FELDMAN, M. (1977) Hydrocortisone affects tumor growth by eliminating precursors of suppressor cells. *J. Immunol.*, **119**, 1563.

STRAUSSER, H. R. & HUMES, J. L. (1975) Prostaglandin synthesis inhibition: effect on bone changes and sarcoma tumor induction in BALB/c mice. *Int. J. Cancer*, **15**, 724.

SYKES, J. A. & MADDOX, I. S. (1972) Prostaglandin production by experimental tumours and effects of anti-inflammatory compounds. *Nature (New Biol.)*, **237**, 59.

TASHJIAN, A. H., VOELKEL, E. F., MCDONOUGH, J. & LEVINE, L. (1975) Hydrocortisone inhibits prostaglandin production by mouse fibrosarcoma cells. *Nature*, **258**, 739.

WEBB, D., BRAUN, W. & PLESCIA, O. J. (1972) Antitumor effects of polynucleotides and theophylline. *Cancer Res.*, **32**, 1814.

WEBB, D. R. & JAMIESON, A. T. (1976) Control of mitogen-induced transformation: characterization of a splenic suppressor cell and its mode of action. *Cell. Immunol.*, **24**, 45.

WEBB, D. R. & LIU OSHEROFF, P. (1976) Antigen stimulation of prostaglandin synthesis and control of immune responses. *Proc. Natl Acad. Sci., USA*, **73**, 1300.

WINCHURCH, R. A., FOSCHI, G. V. & WALZ, D. T. (1974) Inhibition of the lymphocyte-mediated cytotoxic reaction by anti-inflammatory drugs. *J. Reticuloendothel. Soc.*, **15**, 112.